# Epidemiology of *Blastocystis* infection from 1990 to 2019 in China

**DOI:** 10.1186/s40249-020-00779-z

**Published:** 2020-12-30

**Authors:** Chao-Qun Ning, Zhu-hua Hu, Jun-hu Chen, Lin Ai, Li-Guang Tian

**Affiliations:** 1 Chinese Center for Disease Control and Prevention, National Institute of Parasitic Diseases, Shanghai, 200025 People’s Republic of China; 2 Key Laboratory for Parasitology and Vector Biology, MOH of China, WHO Collaborating Center for Tropical Diseases, National Center for International Research On Tropical Diseases, Shanghai, 20025 People’s Republic of China; 3 Nanchang Center for Disease Control and Prevention, Nanchang, 330038 People’s Republic of China

**Keywords:** *Blastocystis*, Epidemiology, Prevalence, Subtype, Diversity, China

## Abstract

**Background:**

*Blastocystis* is ubiquitous presence in animals and humans worldwide and has a high level genetic diversity. The aim of this study was to conduct a summary of *Blastocystis* prevalence, subtypes (STs) in humans and animals in China and depict their distribution.

**Methods:**

We searched for the articles related to epidemiology of *Blastocystis* in humans and animals throughout China which published from January 1, 1990, to July 31, 2019 in the following databases: PubMed, China National Knowledge Infrastructure (CNKI) and Wanfang database. The keywords were *Blastocystis* and one of the following ones: STs, subtypes, distribution, epidemiology, prevalence, infection, molecular, geographic, intestinal parasites, genetic diversity and characterization.

**Results:**

In recent years, various molecular epidemiological studies have been carried out in some provinces/regions of China to identify subtypes of *Blastocystis*. Infants and young children, school students, hospitalized diarrhea patients, HIV/AIDS patients, tuberculosis patients, and cancer patients as respondents had been included. ST1–ST7 and ST12 were the main subtypes in Chinese population. Moreover, surveys of *Blastocystis* infection in animal were also conducted in some provinces of China. A variety of animals were investigated including pigs, cattle, sheep, yak, giant panda, and crested ibis (*Nipponia nippon*) with the main subtypes of ST1–ST8, ST10, ST12–ST14.

**Conclusions:**

In recent years, some provinces/regions in China have conducted various molecular epidemiological studies to identify the *Blastocystis* subtypes. It is important to focus on new subtypes and mixed subtypes of infection, while increasing data on ribosomal alleles. We encourage the scientific community to start research on humans and surrounding animals (including domestic and wild animals) to better understand the possibility of *Blastocystis* transmission between humans and animals. We call for action among researchers studying intestinal parasitic diseases (*Blastocystis*), start drawing the subtype of *Blastocystis* and increase the subtype related to its clinical symptoms.

## Background

*Blastocystis* is widely distributed throughout the world. It is an anaerobic intestinal parasite that can infect humans and a variety of animals [1, 2]. *Blastocystis* is the most common intestinal protozoa in human fecal specimens, which probably due to its rapid propagation and survival ability in different hosts such as humans and animals. Similar to the transmission route of some intestinal protozoans, the route of *Blastocystis* in humans and animals is via fecal-oral transmission, such as through contaminated water and food [3]. Based on gene analysis of small subunit of the ribosomal RNA (SSU-rRNA), wide genetic diversity is observed within *Blastocystis*, and multiple subtypes (STs) have been reported [4]. Currently, at least 17 subtypes are known, of which ST1 to ST8 and ST12 have been reported in humans [5, 6]; ST9 was exclusively identified in humans; and ST10–ST17 were identified only in animals [7]; novel subtypes are still being discovered [8]; and mixed-subtype infections of *Blastocystis* occurs [9]. ST1–ST4 commonly occur in humans, but ST4 is only reported in European region [10].

Clinical manifestations of *Blastocystis* are very diverse, including acute or chronic diarrhea, abdominal pain, nausea, anorexia, bloating, fatigue and flatulence [11, 12], along with allergy [13]. The prevalence of *Blastocystis* is high. Obviously, this parasite has a certain impact on human health, but its role in human health and disease is still uncertain [14]. A high rate of asymptomatic carriers exist in *Blastocystis* infections, and it is still uncertain whether the clinical symptoms of *Blastocystis* infection are related to a specific subtype or several subtypes of *Blastocystis*; or whether it is colonized by multiple parasites, causing the pathogenicity in *Blastocystis* under strong debate [2, 3]. But, Clark proposed that different subtypes may have different pathological potentials [15]. Kaneda et al. suggested that ST1, ST2 and ST4 may be related to gastrointestinal symptoms [16]. *Blastocystis* ST1 is regarded as a pathogenic subtype and associated with irritable bowel syndrome diarrhoea (IBS-D) [17]. *Blastocystis* ST3 is considered to be virulent, which not only increases the pathogenicity of this parasite, but also increases the level of IgE in the serum, thereby causing allergies [13]. Jimenez et al. [3] proposed that the pathogenicity of *Blastocystis* is still controversial for many reasons, for example, the high proportion of asymptomatic carriers, host susceptibility, differences in intestinal microbiota and different pathogenic potentials of different *Blastocystis* subtypes. The prevalence of *Blastocystis* is high in some areas of China, which is mainly attributed to use of earthen toilets or manure pits, contact with animal, drinking unboiled water directly and poor hygiene [18, 19]. *Blastocystis* can colonize the intestines of humans, domestic animals (cattle, sheep, goats and pigs) and wild animals, which has been observed in many provinces in China. Therefore, we describe herein our summary of the studies about epidemiology of *Blastocystis* in humans and animals throughout China, and aim to depict the prevalence of *Blastocystis* in different provinces, display the distribution of *Blastocystis* subtypes among different hosts (humans and animals).

## Methods

### Search strategy

Geographically, the study domain was restricted to research in China. Studies reported in English and Chinese were selected. The information in the article includes whether *Blastocystis* and epidemiology (infection status and/or subtype) in humans or animals are mentioned.

**Fig. 1:**
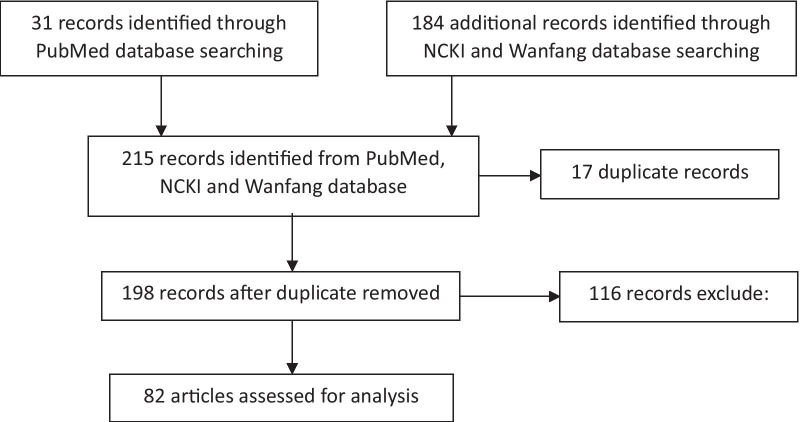
Flow diagram, literature search and screening process

Both PubMed and China National Knowledge Infrastructure (https://www.cnki.net/) and Wanfang (http://www.wanfangdata.com.cn/index.html) database were used to find potentially eligible articles. The articles about epidemiology of *Blastocystis* were searched in humans and animals throughout China, which were published from January 1, 2010, to July 31, 2019. There included keywords *Blastocystis* and one of the following keywords: STs, subtypes, distribution, epidemiology, prevalence, infection, molecular, geographic, intestinal parasites, genetic diversity and characterization. Duplicate studies from the three databases were removed. We excluded conference abstract papers, case reports, case series and review articles. For the same survey, multiple results may be published in different forms, and we select articles with complete results. A total of 215 articles were found, and only 82 of them met the above criteria (Fig. 1). More details of the 82 articles were displayed in Additional file 1: Table S1, including title, author, journal.

### Information extraction and analysis

Two investigators independently screened abstracts, full-text articles, performed data extraction. Data were extracted by the first investigator from the included articles, evaluated by the second investigator and final evaluation was conducted by the third investigator. The characteristics were extracted from each study, including the surveyed province, number of samples, number of samples positive for *Blastocystis*, host, detection method, subtypes, number of samples per subtype, article title, first author and year of publication. We extracted relevant information from each article that met the inclusion criteria of this study.

According to the different detection methods of *Blastocystis* in the study, the overall infection rate in China and the infection rate in each province were calculated, and revealed the distribution of subtypes in different provinces in China. At the same time, we could determine the distribution of subtypes in humans and different kinds of animals.

## Results

### Distribution of investigation of *Blastocystis* infection

For the study of *Blastocystis* infection in humans, we divide the humans into the general population, students, children, hospitalized or outpatients, diarrhea cases, and people with HIV or tuberculosis and other diseases. We have summarized the infection of *Blastocystis* in humans and animals, shown in Tables 1 and 2.

**Table 1:** Infection and characteristics of *Blastocystis* in humans

No	Author, year, reference number	Study area (years of the survey)	Study design	Age range /mean or median	Sex (*n*)	Participants	No. positive (prevalence)	Method of diagnosis	Primer sequence (amplification length)	Subtypes (*n*)	Clinical manifestation (*n*/*N*)	*Blastocystis* subtypes in patients with diarrhea (*n*)
1	Li et al. 2007, [20]	Shanghai (2006)	Cross-sectional observational study	2–96 years/–	NA	1505 people living in villages	29 (1.93%)	PCR	NA	ST1 (6), ST2 (1), ST3 (17), ST6 (1), ST1 + ST3 (2), Unknown (2)	NA	NA
2	Li et al. 2007, [20]	Eryuan county, Yunnan (2005)	Cross-sectional observational study	5–57 years/–	NA	407 people from villages	75 (18.43%)	PCR	NA	ST1 (22), ST2 (6), ST3 (38), ST1 + ST3 (5), ST2 + ST3 (1), Unknown (3)	NA	NA
3	Li et al. 2007, [20]	Yongjia county, Zhejiang (2006)	Cross-sectional observational study	4 months to 90 years/–	NA	170 in-patients	10 (5.88%)	PCR	NA	ST1 (3), ST2 (1), ST3 (6)	NA	NA
4	Gong and Liu 2019, [21]	Mengla county, Yunnan (2014)	Cross-sectional observational study	21–72 years/–	NA	289 Yao people from villages	13 (4.50%)	PCR	NA (260 bp)	ST1 (3), ST3 (8), ST4 (1), Unknown (1)	Diarrhea (6/29^a^), abdominal pain (2/29^a^)	NA
5	Zhou et al. 2019, [22]	Rural permanent population in Henan (2015)	Cross-sectional observational study	1–101 years/ 38.95 years	Male (3191), female (3515)	6706 residents of Qinba Mountains Ecological Zone	3 (0.04%)	Iodine	NA	NA	NA	NA
6	Zhang et al. 2016, [23]	Kunming city, Yunnan (2014–2015)	Case-control study	Diarrhea cases (1121): 0–6 months (186), 6–12 months (263), 1–2 years (269), 2–5 years (132), 5–65 years (244), > 65 years (27)	Diarrhea cases (1121): male (559), female (562). Non-diarrhea cases (319): male (165), female (154)	1121 diarrhea cases and 319 non-diarrhea cases from hospitals	Diarrhea cases: 47 (4.19%), non-diarrhea cases: 11 (3.44%)	PCR	Forward primer (5′-CGAATGGCTCATTATATCAGTT-3′), reverse primer (5′-TCTTCGTTACCCGTTACTGC-3′), (1100 bp)	Diarrhea cases: ST1 (46), ST2 (1) non-diarrhea cases: ST1 (11)	Diarrhea cases: vomiting (263/1121), dehydration (64/1121)	Diarrhea cases: ST1 (46), ST2 (1)
7	He 2013, [24]	Bama Yao autonomous county, Guangxi (2011)	Cross-sectional observational study	2–80 years/–	Male (253), female (244)	497 local residents	215 (43.26%)	Improved acid ether centrifugal sedimentation /PCR^b^	Forward primer (F): 5′-GAAGGACTCTCTGACGATGA-3′, reverse primer (R): 5′-GTCCAAATGAAAGGCAGC-3′ (351 bp); F: 5′-ATCAGCCTACAATCTCCTC-3′, R: 5′-ATCGCCACTTCTCCAAT-3′ (650 bp); F: 5′-TAGGATTTGGTGTTTGGAGA-3′, R: 5′-TTAGAAGTGAAGGAGATGGAAG-3′ (526 bp)	ST1 (25), ST6 (1), ST1 + ST6 (1), Unknown (78)	NA	NA
8	Yang 2011, [25]	Beihai city and Qinzhou city, Guangxi (2010)	Cross-sectional observational study	6 months to 91 years	Male (702), female (664)	1366 rural permanent population	360 (36.35%)	Improved acid ether centrifugal sedimentation /PCR^c^	Forward primer (F): 5′-GAAGGACTCTCTGACGATGA-3′, reverse primer (R): 5′-GTCCAAATGAAAGGCAGC-3′ (351 bp); F: 5′-ATCAGCCTACAATCTCCTC-3′, R: 5 -ATCGCCACTTCTCCAAT-3′ (650 bp); F: 5′-TAGGATTTGGTGTTTGGAGA-3′, R: 5′-TTAGAAGTGAAGGAGATGGAAG-3′ (526 bp)	ST1 (12), ST3 (2), ST4 (1), ST6 (1), ST7 (3), ST1 + others (6), Unknown (12)	NA	NA
9	Wang 2015, [26]	Guangxi (2013–2014)	Cross-sectional observational study	HIV/AIDS patients: 21–85 years/mean 48.6, general population: 18–81 years/mean 46.6	HIV/AIDS patients: male (216), female (69), general population: male (101), female (49)	285 HIV/AIDS patients, 150 general population	HIV/AIDS patients: 59 (20.70%), general population: 38 (25.33%)	Improved acid ether centrifugal sedimentation	NA	NA	NA	NA
10	Tian et al. 2012, [27]	Fuyang city, Anhui (2008)	Case-control study	HIV positives: 6–65 years/mean 42.8 years, HIV negative individuals: 6–65 years/mean 41.5 years	HIV positives: 143 males and 159 females, 303 HIV negative Individuals: 144 males and 159 females	302 HIV positives, 303 HIV negative Individuals	HIV positives: 49 (16.23%), HIV negative Individuals: 67 (22.11%)	In vitro culture	NA	NA	NA	NA
11	Teng et al. 2018, [28]	Tengchong city, Yunnan (2016–2017)	Cross-sectional observational study	10–74 years/mean 40.4 years	HIV positives: male (157), female (167)	324	12 (3.70%)	PCR	Forward primer: 5′-GGAGGTAGTGACAATAA-ATC-3′; reverse primer: 5′-ACTAGGAATTCCTCGTTC-ATG-3′ (1100 bp)	ST1 (3), ST3 (2), ST4 (3), ST7 (3), ST12 (1)	Diarrhea (3/12)	ST1 (2), ST12 (1)
12	Li et al. 2015, [29]	Gushi county, Henan (2012)	Cross-sectional observational study	≤ 60 years old (153), > 60 years old (188)/median 62 years	Male (249), female (120)	369 Patients with pulmonary TB (PTB) undergoing anti-*Mycobacterium* tuberculosis treatment	22 (5.96%)	In vitro culture	NA	NA	NA	NA
13	Zhang et al. 2017, [30]	Harbin city, Heilongjiang (2016–2017)	Cross-sectional observational study	25 to 84 years	Male (220), female (161)	381 cancer patients: lung (90), stomach (88), colorectal (49), liver (47), esophagus (29), breast (28), hematologic (22), other types of cancer (28)	27 (7.09%)	PCR	NA (600 bp)	ST1 (12), ST3 (15)	Diarrhea (14/27)	ST1 (8), ST3 (6)
14	Hu et al. 2015, [31]	Nanning city, Guangxi (2013–2014)	Cross-sectional observational study	5 to 79 years	Male (378), female (305)	683 tumor patients: Digestive system (228), respiratory system (189), urinary system (128), nervous system (87), other tumor patients (51)	Smear: 29(4.25%), improved acid ether centrifugal sedimentation: 83 (12.15%)	Smear/improved acid ether centrifugal sedimentation	NA	NA	NA	NA
15	Hu et al. 2017, [32]	Nanning city, Guangxi (2016)	Cross-sectional observational study	< 42 years old (310), ≥ 42 years old (603)	Male (367), female (546)	913 patients with chronic diseases	Smear: 30 (3.29%), improved acid ether centrifugal sedimentation: 137 (15.01%)	Smear/improved acid ether centrifugal sedimentation	NA	NA	Diarrhea (80), abdominal pain (71), anorexia (51), nausea and vomiting (34), wasting (42), fever (27)	NA

*NA* not available, – not applicable

^a^Number of symptomatic cases among patients infected with *Blastocystis* at China-Myanmar border

^b^105 samples of positive samples were cultured and PCR tested

^c^37 of the positive samples were tested by PCR

**Table 2:** Infection and characteristics of *Blastocystis* in animals in China

No	Author, year, reference number	Study area (years of the survey)	Hosts (age or gender)	No. positive	Prevalence (%)	Method of diagnosis	Sequence of primers (Product size)	Subtypes (*n*)
1	Nong et al. 2012, [33]	Guangxi (2010)	150 Rhesus monkeys (2.5–4 years)	29	19.33	Improved acid ether centrifugal sedimentation	NA	NA
2	Zhao et al. 2017, [34]	Qinling Mountains, Shaanxi, (2015–2016)	497 Wild animals: 127 Nonhuman primates, 158 Artiodactyla, 18 Perissodactyla, 3 Proboscidae, 11 Marsupialia, 135 Aves, 45 Carnivora (NA)	Wild animals: 200/497, Nonhuman primates: 96/127, Artiodactyla: 89/158, Perissodactyla: 4/18, Proboscidae: 0/3, Marsupialia: 8/11, Aves: 3/135, Carnivora: 0/45	Wild animals: 40.24, Nonhuman primates: 75.59, Artiodactyla: 56.33, Perissodactyla: 22.22, Proboscidae: 0.00, Marsupialia: 72.73, Aves: 2.22, Carnivora: 0.00	PCR	5′-GGAAGCTTATCTGGTTGAT CCTGCCAGTA-3’ 5′-GGGATCCTGATCCTTCCG CAGGTTCACCTAC-3′ (1800 bp); 5′-GGAGGTAGTGACAATAAATC-3’ 5′-ACTAGGAATTCCTCGTTCATG-3′ (1100 bp)	ST1 (32), ST2 (13), ST3 (12), ST5 (2), ST10 (77), ST12 (5), ST13 (37), ST14 (17), ST18^*^ (1), ST19* (1), ST20* (1), ST21* (1), ST22* (1)
3	Zhang et al. 2015, [35]	Zhouzhi county, Xi’an city, Shaanxi (2008)	63 captive breeding crested ibis (1:1 male to female ratio)	6/63	9.52	Smear/Iodine	NA	NA
4	Deng et al. 2019, [36]	Sichuan (2017–2018)	81 giant pandas (< 1.5 years: 4, 1.5–5.5 years: 23, > 5 years: 54. Male 31, female 50, 23 red pandas, 64 birds	Giant pandas: 10/81, red pandas: 2/23, birds: 7/64	Giant pandas: 12.35, red pandas: 8.69, birds: 10.94	PCR	Forward primer: 5′-GGAGGTA GTGACAATAAATC-3′, reverse primer: 5′-TGCTTTCGCACTTG TTCATC-3′, (600 bp)	Giant pandas: ST1 (10), red pandas: ST1 (2), birds: ST8 (7)
5	Wang et al. 2018, [37]	Heilongjiang, Liaoning and Jilin (2015–2017)	1080 mammals, 185 birds	Mammals: 41/1080, birds: 13/185	Mammals: 3.80, birds: 7.02	PCR	Forward primer: 5′-GGAGGTA GTGACAATAAATC-3′, reverse primer: 5′-TGCTTTCGCACTTG TTCATC-3′, (600 bp)	Mammals: ST1 (5); ST3 (3); ST4 (13); ST7 (1); ST10 (13); ST13 (4); ST14 (2), birds: ST6 (8); ST7 (5)
6	Wang et al. 2018, [38]	Hionglongjiang (2010–2016)	Pig: 68, cattle: 147, sheep: 109 and goats:13	Pig: 6/68, cattle: 14/147, sheep: 6/109 and goats: 0/13	Pig: 8.82, cattle: 9.52, sheep: 5.50	PCR	Forward primer: 5′-GGAGGTA GTGACAATAAATC-3′, reverse primer: 5′-TGCTTTCGCACTTG TTCATC-3′, (600 bp)	Pig: ST5 (6), cattle: ST3 (2), ST10 (10), ST14 (2), sheep: ST1 (1), ST5 (1), ST10 (3), ST14 (1)
7	Song et al. 2017, [39]	Shaanxi (2014–2016)	Dairy goats: 362, meat goats: 193, cashmere goats: 234	Dairy goats: 196/362, meat goats: 78/193, cashmere goats: 184/234	Dairy goats: 54.14, meat goats: 40.41, cashmere goats: 78.63	PCR	Forward primer: 5′-GGAGGTA GTGACAATAAATC-3′, reverse primer: 5′-TGCTTTCGCACTTG TTCATC-3′, (600 bp)	Dairy goats: ST1 (1), ST3 (1), ST5 (28), ST10 (132), ST14 (33), novel (1). Meat goats: ST10 (37), ST14 (41). Cashmere goats: ST4 (9), ST5 (3), ST10 (123), ST14 (49)
8	Zhu et al. 2017, [40]	Daqing city, Qiqihar city, Harbin city, Heilongjiang (2013–2014)	526 cattle: < 3 months (*n* = 69), 3–12 months (*n* = 61), and aged > 12 months (*n* = 66)	Cattle:54/526. Daqing city: 9/140, Qiqihar city: 0/190, Harbin city: 45/196	Cattle (10.27). Daqing city: 6.43, Qiqihar city: 0.00, Harbin city: 22.96	Nested PCR	RD3 (5′-GGGATCCTGATCCTTC CGCAGGTTCACCTAC-3′) and RD5 (5′-GGAAGCTTATCTGGTT GATCCTGCCAGTA-3′) (1780 bp) 2F (55 -GGGATCCTGATCCTTC GT-3′) and 2R (5′-AGCTTTTT AACTGCAACAACG-3′), (600 bp)	Daqing city: ST10 (3), ST14 (6), Harbin city: ST4 (2), ST5 (1), ST10 (38), ST14 (4)
9	Ren et al. 2019, [41]	Qinghai (2016–2017)	1027 yaks (≤ 6 months: 48, > 6 months: 979)	Yaks: 278/1027	27.07	Nested PCR	NA	ST10 (170), ST12 (38), ST14 (70)
10	Li et al. 2019, [42]	Zhejiang, Anhui, Shanghai, Jiangsu, Shandong, Jiangxi (2015–2018)	346 cats (151 males and 195 females. ≤ 12 months (60) and > 12 months (286)	2/346, *Blastocystis* was only observed in Lu’an, Anhui (22.2%, 2/9)	0.57	PCR	NA	ST1 (2)
11	Xiao et al. 2019, [43]	Enshi county, Hubei (2017)	69 flying squirrels (3 fecal samples per animal)	Fecal samples: 63/207, flying squirrels 21/69	30.43	Nested PCR	RD5: 5′-GGAAGCTTATCTGGTTG ATCCTGCCAGTA-3′, RD3: 5′-GG GATCCTGATCCTTCCGC AGGTT CACCTAC-3′, (1800 bp); RDII5: 5′-GGAGGTAGTGACAAT AAATC-3′, RDII3: 5′-ACTAGGAA TTCCTCGTTCATG-3′, (1100 bp)	ST1 (24), ST3 (12), ST13 (27)

*NA* not available

### Risk factors of *Blastocysti*s infection in humans

The risk factors of *Blastocystis* infection are diverse. Several studies have shown that not washing hands after going to the toilet, drinking unboiled tap water, eating outside for a long time, raising poultry or livestock, low immune function, poor nutritional status, female, body mass index < 19, anemia and barefoot working in farm are the risk factors that cause *Blastocystis* infection [21, 28, 29, 32, 44, 45]. In addition, suffering from some underlying diseases such as *Helicobacter pylori* infection and hepatitis B are also risk factors for *Blastocystis* infection [46].

#### Infection status and subtypes of Blastocystis in general population

In general population, the infection status and subtype distribution of *Blastocystis* in China are shown in Table 1. The infection rate of *Blastocystis* in the population has regional differences. For example, surveys in different regions of Guangxi found that the infection rate of *Blastocystis* in Bama Yao Autonomous County was 43.26%, and in Beihai and Qinzhou was 36.35% [24, 25]. The methods used in the two studies were the same and the subjects were the local general population. Yan et al. identified that the subtypes of *Blastocystis* in humans were ST1–ST3. ST3 was the main subtype (40.0%), followed by ST1 (37.1%), the mixed subtype of ST1 and ST3 accounted for 14.3%, and unknown subtypes have also been found [47].

#### Blastocystis infection in students and children

There are few studies focus on *Blastocystis* infection in students. The infection rate of *Blastocystis* in primary and university students from Jiangxi was 1.10% and 10.09%, respectively [48, 49]. The prevalence of *Blastocystis* in college students in Guangxi was 14.93% [50]. Another study in Guangxi found that infection subtypes include ST1, ST3, ST4, ST6 and ST7, among which ST3 was the main subtype (32.08%) [51].

A study found that children in Jiangxi Province have a higher infection rate (35.9%) and have symptoms such as diarrhea and recurrent abdominal pain [52]. The infection rate of children with diarrhea in Yunnan (3.1%) and Fujian (8.94%) was lower than that in Jiangxi [53, 54]. Cao et al. performed fecal microscopy on children in Shenzhen Children’s Hospital and found that the infection rate of *Blastocystis* was low (0.4%) [55].

#### Infection status and subtype of Blastocystis in inpatients or outpatient without distinction of disease

Some investigations related to *Blastocystis* infections were conducted in the hospitals, and the subjects were hospitalized or outpatients. These patients were randomly selected from the hospital. The infection rate of patients in the First Affiliated Hospital of Guangxi Medical University was 22.78%, and the infection rate was significant different in gender. The disease of these patients was not clear [56]. The study found that the infection rate of *Blastocystis* in hospitals in Nanning, Guangxi has little change over time. The prevalence of patients were 16.27% and 16.77%, respectively in 2005 and 2013 [57].

#### Blastocystis infection and its subtypes in patients with diarrhea

*Blastocystis* was one of the common pathogens in patients with diarrhea. The main manifestations of patients with *Blastocystis* include abdominal pain or diarrhea, followed by fatigue and anorexia. Some researchers have found that these gastrointestinal symptoms may be related to colitis [58]. Diarrhea patients have different infection rates in different seasons, and the subtype was mainly ST1 [23, 59]. There are few studies on the association between the subtypes of *Blastocystis* and clinical manifestations, some of which are related to the subtypes of *Blastocystis* in patients with diarrhea (Table 1).

#### Blastocystis infection in patients with underlying diseases

There are few studies on the co-infection of *Blastocystis* and underlying diseases (HIV/AIDS patients, tuberculosis patients, cancer patients and chronic disease patients). The prevalence, clinical manifestation and subtypes of *Blastocystis* in patients with HIV/AIDS, pulmonary TB, cancer and chronic diseases show in Table 1. *Blastocystis* and HIV co-infection were studied in Yunnan, Anhui and Guangxi provinces. Studies have suggested that *Blastocystis* infection increases the level of IL-2 in HIV-infected persons, changes the Th1/Th2 balance, and accelerates the conversion of HIV infection to AIDS [60]. In China, ST12 infection was first detected in AIDS patients in 2018 [28]. There was no difference in the infection of *Blastocystis* in tuberculosis patients (6.2%) and the healthy group (7.6%) [44]. The detection of cancer patients found that the infection rate of *Blastocystis* in lung, stomach, colorectal cancer patients was higher than that of other cancer patients, and the infection rate of cancer patients was significantly higher than that of the general population (malignant tumors: 43.24%, non-malignant tumors: 22.59%, 19.70% of the general population) [61]. The symptoms of diarrhea in cancer patients may be related to ST1 [30]. The infection rate of *Blastocystis* in patients with chronic disease was 18.29%, and clinical symptoms such as abdominal pain, diarrhea, and vomiting may occur [32].

### Distribution and infection of *Blastocystis* in animals

There is a list of the distribution and genetic diversity of *Blastocystis* in different animals including non-human primates, birds, and mammals in China. Some provinces have performed genotyping of animal *Blastocystis* (Table 2). There are significant differences in the prevalence of *Blastocystis* in pigs in different regions and age groups [34]. The infection rate of *Blastocystis* in cats was low. Among the 346 cats surveyed in six provinces, only 2 cats were found to be infected with *Blastocystis* in Lu’an, Anhui Province [42]. Among animal *Blastocystis* isolates, the potential zoonotic subtype including ST1, ST3 and ST5 accounts for 38.5% [38]. At the same time, insects could also be infected, and the body surface and digestive tract of cockroach and housefly can be infected with *Blastocystis* [62].

## Discussion

Based on the above information, we found that the infection of *Blastocystis* in different populations and regions is different. The infection rate of primary school students was lower than that of college students [48, 49]. It may be that under the management of parents and teachers, primary school students have developed good hygiene habits, such as washing hands frequently. The investigations of *Blastocystis* infection in hospitalized patients found that the infection rate in Guangxi was higher than that in other regions. This may be due to the help of doctors after local residents developed symptoms such as diarrhea, or different detection methods used in the study. The specific reasons need to be studied in depth [63]. Studies on patients with diarrhea have shown that ST1 is related to clinical symptoms such as diarrhea and has potential pathogenicity. The main subtype of diarrhea patients is ST1, and the main subtype of asymptomatic *Blastocystis* infection is ST3 [23, 30, 45]. CD4 + cell count ≤ 500 cells/μl, and an HIV-RNA viral load > 50 copies/ml were the influencing factors of *Blastocystis* infection in HIV-seropositive individuals [64].

Considering the subtype of *Blastocystis* in humans and animals, this study found that ST1–ST7 and ST12 were present in the sample of humans in China [28, 63, 65], of which a large number were typed most frequently as ST1, ST2 and ST3 [21, 23, 51, 66], including mixed subtypes of ST1 and ST3, ST1 and ST2, and ST2 and ST3 [20], followed by other subtypes in minor percentages. Foreign studies have found that ST1–ST9 of *Blastocystis* can infect humans [67]. However, it has not been found that ST8 and ST9 can infect humans in China. This study found that ST1–ST8 and ST10, as well as ST12–ST14 were present in the sample of animals in China (Table 2). Some foreign studies have found that ST1–ST17 can infect animals [67]. However, ST9, ST11, ST15–ST17 have not been found to infect animals in China. In China, ST10 predominates in animal infection [39–41, 68, 69], followed by ST5 [34]. This is different from some foreign studies. An Italian study found that the subtype of *Blastocystis* infection in dogs was ST3 [70]. The subtype of *Blastocystis* infection in animals from the United States was mainly ST8 (20.6%), followed by ST6 (17.3%) and ST5 (15.9%) [3]. Because ST1–ST8 subtypes can infect both humans and animals, there are studies abroad to explore the possibility of the transmission of *Blastocystis* between humans and animals. In 2019, a report in Lebanon believed that *Blastocystis* has a potential risk of transmission from livestock to its contacts. The study found that ST1, ST2, ST3 infected in cows and people who have been in contact with cows, and the sequence of ST3 is exactly the same between cows and their contacts [71]. Few studies conducted in this research field in China, and there is a lack of corresponding data. The most recent epidemiological data of *Blastocystis* and its subtypes are limited to reports from a few provinces in China [40, 53, 59], and most of these reports come from research conducted by certain institutions. We are considering summarizing the distribution of *Blastocystis* and its subtypes in various provinces in China. However, there are no data available in many provinces/regions. The fact that most of the research comes from a few provinces may be related to the fact that there are more investigators and enough attention. This shows that in underrepresented provinces, more investigators and more attention are needed to infer the true distribution of *Blastocystis* in different regions of China.

There are several limitations in our study. Firstly, most of our included studies were cross-sectional studies where selection bias may have occurred, for example, many research subjects are selected from hospitals, schools, etc. Secondly, the detection methods of *Blastocystis* in each study are different, and the primers and primer lengths are different when performing polymerase chain reaction (PCR) detection. Thirdly, some provinces have conducted fewer studies or only conducted studies in a certain urban area, and the majority of province no research on *Blastocystis* has been conducted, so some data are less representative. Finally, in addition to real differences, the possible reasons for the different infection rates among different regions may be attributed to the large time span of this study (1990–2019) and the possible differences in the results of the researcher’s identification through microscopy.

## Conclusions

In recent years, various molecular epidemiological studies have been conducted in some provinces/regions of China to identify the subtypes of *Blastocystis*. We believe that it is important to focus on new subtypes and mixed subtypes of infection, while increasing data on ribosomal alleles. In addition, the relationship between *Blastocystis* subtypes and clinical symptoms should be studied. Finally, we should pay attention to the people and surrounding animals (including domestic and wild animals) to better explore the possibility and means of transmission of *Blastocystis* between humans and animals.

## Supplementary information

**Additional file 1:** Articles that meet the criteria.

## Data Availability

All datasets are presented in the main paper.

## Abbreviations

ST: Subtype
CNKI: China National Knowledge Infrastructure
HIV: Human immunodeficiency virus
SSU-rRNA: Small Subunit Ribosomal RNA
IBS-D: Irritable bowel syndrome diarrhoea
PCR: Polymerase chain reaction
