# Supplementary material for: Epidemiology of Blastocystis infection from 1990 to 2019 in China
Source: Infect Dis Poverty. 2020 Dec 30;9:168. doi: 10.1186/s40249-020-00779-z (PMC7772921; doi:10.1186/s40249-020-00779-z)
Supplement: Supplementary file 1 — Additional file 1: Articles that meet the criteria. [file 40249_2020_779_MOESM1_ESM.docx]

| **ID** | **Title** | **Author** | **Provenance** |
| --- | --- | --- | --- |
| 1 | PCR-based Genotype Classification of Blastocystis hominis Isolates from College Students of Guangxi | ZHAN Ting-zheng, LIU Teng, SHI Huan-huan, HE Shan-shan, Yan Hui, LIU Deng-yu | Chinese Journal of Parasitology and Parasitic Diseases |
| 2 | The epidemiological characteristics and influencing factors for Blastocystis hominis infection among human immunodeficiency virus seropositive individuals in Tengchong of Yunnan Province | TENG Xue-jiao, CHU Yan-hong, ZHAI Cheng-cheng, YU Ying-fang, CAI Yu-chun, CHEN Shao-hong, AI Lin, TIAN Li-guang, CHEN Jia-xu | Chinese Journal of Parasitology and Parasitic Diseases |
| 3 | Investigation of infection in patients with chronic diseases | HU Ying, LI Yan-wen, LIU Xiao-quan, SHI Ji-ying | Chinese Journal of Health Laboratory Technology |
| 4 | Investigation on the infection of Blastocystis hominis among college students and hospital attendees in Nanning | Wang Jing, Liu Teng, Shi Huanhuan, Wang Ge, Liu Dengyu, Liu Xiaoquan, Lu Zuochao, Shen Jiqing | Journal of Applied Preventive Medicine |
| 5 | The infection status of Blastocystis hominis in the 2010 freshmen of Guilin Medical College | Mo Gang, Yang Dongyu, Zeng Lingying, Nong Zijun, Li Yunping | Chinese Journal of School Health |
| 6 | Clinical investigation of infectious status of Blastocystis hominis in Guangxi | TIAN Chun-lin, HE Deng-xian, WAN Xiao-ling | JOURNAL OF APPLIED PREVENTIVE MEDICINE |
| 7 | Investigation of infectious status of Blastocystis hominis in 1 354 outpatients | Jin Qunxin, Yu Kaimin, Tang Lianfeng, Tian Chunlin, Lu Zuochao | CHINA TROPICAL MEDICINE |
| 8 | Research on the infection status of Blastocystosis hominis in patients with malignant tumour | HU Ying, SONG Xiang-qun, LI Yan-wen, SHI Huan-huan | Chinese Journal of Health Laboratory Technology |
| 9 | Detection of Blastocystis hominis in children with abdominal pain and diarrhea: an analysis of 28 cases | Wang Ling, Su Shuilian, Chen Guifeng | THE JOURNAL OF CLINICAL PEDIATRICS |
| 10 | Study on children's chronic diarrhea and Blastocystis hominis infection in Jiangxi area | Zhao Li, Dai Shuming, Chen Fanggen | Medical Information |
| 11 | Epidemiological investigation on infection of blastocystis hominis in various populations in Guilin | Nong Zijun, Mo Gang, Li Yunping, Jiang Liping, Shi Huanhuan | CHONGQING MEDICINE |
| 12 | INFECTION STATUS OF BLASTOCYSTIS HOMINIS IN DIARRHEAL PATIENTS AND ITS REPRODUCTION | Da Rong, Qiao Jiying, Yang Junhua, Li Chen | CHINESE JOURNAL OF PARASITIC DISEASE CONTROL |
| 13 | Investigation of infectious status of blastocystis hominis in Guangxi | Liu Ying, Qian Cheng, Chen Xiangren, Zeng Dequan, He Aijuan, Yang Yuanhua, Lu Zuochao, Liu Dengyu | JOURNAL OF APPLIED PREVENTIVE MEDICINE |
| 14 | Preliminary investigation on the infection of Blastocystis hominis in children in Shenzhen Children's Hospital | Cao Ke, Luo Xiaojuan, Wang Danlin, Zhang Yi, Tang Dan, Chen Yunsheng, Ma Dongli | Chinese Journal of Clinical Laboratory Science |
| 15 | Prevalence and distribution of Blastocystis hominis in Qinghai Province | He Duolong | QINGHAI MEDICAL JOURNAL |
| 16 | INVESTIGATION OF COLLEGE STUDENT INFECTION WITH BLASTOCYSTIS HOMINIS IN GUANGXI MEDICAL UNIVERSITY | Liu Teng, Liu Xiaoquan, Shi Huanhuan, He Shanshan, Wang Ge, Fu Ruijia, Lin Junyi, Luo Baohua, Wei Yi, Chen Xi, Yi Qianyan, Zou Qi | Journal of Guangxi Medical University |
| 17 | Survey epidemiology of Blastocystis hominis infection in Ganzhou | SU Zi-lin, SU Shui-lian, WU Zhong-fa, LIAO Hua, LIAO Yong, XIE Qiong-jun, ZHANG Qing-sheng | JOURNAL OF PATHOGEN BIOLOGY |
| 18 | Investigation on Guilin Rhesus Monkeys Infected with Blastocystis hominis | Nong Zijun, Li Yunping, Mo Gang, Jiang Liping, Li Xinde | Heilongjiang Animal Science and Veterinary Medicine |
| 19 | The infection of Blastocystis hominis among pupils in Nantang Town, Gan County | Cao Gaolu, Wu Guohong, Guo Zengzhu | JOURNAL OF GANNAN MEDICAL COLLEGE |
| 20 | Investigation of the prevalence of Blastocystis hominis infection among people in Nanning | Hu Ying, Li Xueming, Zhang Hongman, Shi Huanhuan | Journal of Parasitic Biology |
| 21 | Investigation on the infection of Blastocystis hominis in various populations in Shanghai, China | ZHANG Xiao-ping, LI Lan-hua, ZHU Qian, FU Ying-hua, MA Xiao-jiang, LV Shan, HONG Guo-bao, HE Yan-yan, WANG Zhen-yu, MA Xing-bao | JOURNAL OF PARASITIC BIOLOGY |
| 22 | Clinical analysis of Blastocystis hominis infection in 1185 patients with chronic diarrhea in 2011 | HU Ying, LI Yan-wen, LU Zuo-chao | China Tropical Medicine |
| 23 | Investigation on the Infection of Blastocystis hominis in Populations in Bama Yao Autonomous County of Guangxi | He Shanshan, Wu Lingyuan, Liu Xiaoquan, Shi Huanhuan, Chen Zhi, Zhang Hui, Pang Caiying, Li Yumei | Chinese Journal of Parasitology and Parasitic Diseases |
| 24 | Investigation on the infection of Blastocystis hominis in part of the populations in Xiangxi Autonomous Prefecture , Hunan | Zhang Kairen, Yang Shengyuan, Peng Haiyan, Duan Jihui | Parasitoses and Infectious Diseases |
| 25 | The infection status and clinical study of Blastocystis hominis in adults with chronic diarrhea in Ganzhou area | Xie Zhijun, Zhang Ruiqi, Huang Wenfeng, Liao Yueguang, Su Shuilian | JOURNAL OF SOUTHERN MEDICAL UNIVERSITY |
| 26 | INVESTIGATING BLASTOCYSTIS HOMINIS INFECTION OF INPATIENTS IN TWO TERTIARY HOSPITALS OF GUANGXI | Wang Beibei, Yan Hui, Shi Huanhuan, Liu Dengyu, Liao Dejun, Liu Xiaoquan, Lu Zuochao | Journal of Guangxi Medical University |
| 27 | Investigation on the distribution of human intestinal parasites in Beilun District, Ningbo City in 1997 | Xu Guofeng, Zhang Qinyong, Li Yueliang | ZHEJIANG JOURNAL OF PREVENTIVE MEDICINE |
| 28 | Survey on Asymptomtic Blastocystis Hominis and Its PCR Identification | Zhang Hongwei, Yan Qiuye, He Lijun, Su Yunpu, Li Wen, Xue Changgui, Wang Dong | JOURNAL OF MEDICAL FORUM |
| 29 | Several newly discovered parasitic diseases in recent years | Chen Yatang | CHINESE JOURNAL OF PRACTICAL INTERNAL MEDICINE |
| 30 | Blastocystis Infection in Pet Dogs in Selected Areas of Anhui and Zhejiang Provinces | Li Wenchao, Wang Kai, Qin Mian, Liu Zonghua, Yuan Gan, Liu Deyi, Gu Youfang | Chinese Journal of Parasitology and Parasitic Diseases |
| 31 | INVESTIGATION OF AN EPIDEMIC OUTBREAK OF BLASTOCYSTISASIS | Wu Guohong, Xiong Yishu, Cao Haolu, Li Guangming, Liu Maozhuang, Zhu Jinglin | CHINESE JOURNAL OF PARASITIC DISEASE CONTROL |
| 32 | Examination and Treatment of the Blastocystis Hominis Infection in the Child Repeatedly Abdominal Pain | Liao Hongqun, Qiu Wei, Wang Ling, Liu Xiaosheng, Su Shuilian | JOURNAL OF GANNAN MEDICAL UNIVERSITY |
| 33 | Investigation on Blastocystis hominis, a primate in Guangzhou | He Jianguo, Zhong Guojuan, Zeng Zhongxing | CHINESE JOURNAL OF ZOOLOGY |
| 34 | Clinical epidemiological investigation of intestinal pathogenic parasite infection in children with diarrhea | Huang Miaohui, Chen Suqing, Wu Bin, Wu Lise, Zhao Ziqing | CHINA PUBLIC HEALTH |
| 35 | ANALYSIS OF 12 MISDIAGNOSTIC CASES ON HYPERTHROIDISM IN ?MIDDLE AND OLD PATIENTS WITH SINGLE SYMPTOM | Su Yunpu, Li Wen, He Lijun, Yan Qiuye, Shi Hefeng, Zhao Kaiyuan | HENAN MEDICAL RESEARCH |
| 36 | Genotype Analysis and Isoenzyme Patterns of Ten Isolates of Blastocystis hominis from Guangxi | Zhan Tingzheng, Shi Huanhuan, He Shanshan, Liu Teng | Chinese Journal of Parasitology and Parasitic Diseases |
| 37 | Investigation of Blastocystis hominis Carried by Patients with Diarrhea | Yang Dongchang, Wu Wenzhe, Liu Min | INTERNATIONAL JOURNAL OF LABORATORY MEDICINE |
| 38 | The flow regulation, clinical observation and pathogenic mechanism of Blastocystis hominis | Su Qingping, Su Jinghua, Xie Manling, Huang Yihong, Feng Yian, Wang Jiyun, Zeng Yuebin, Xiao Zhensheng | CHINESE JOURNAL OF ZOONOSES |
| 39 | Epidemiology investigation of Blastocystis hominis in Huainan areas | Li Chaopin, Wang Jian | JOURNAL OF THE FOURTH MILITARY MEDICAL UNIVERSITY |
| 40 | MIXED INFECTION OF MICROSPORIDIA AND BLASTOCYSTIS HOMINIS AND THE OBSERVATION ON MORPHOLOGY OF THE PATHOGENS | Zhang Ruilin, Liang Chi, Lin Liuwen | CHINESE JOURNAL OF ZOONOSES |
| 41 | INVESTIGATION ON HUMAN INFECTION OF BLASTOCYSTIS HOMINIS IN SICHUAN PROVINCE. | Liu Changhua, Xie Hong, Zheng Defu | MODERN PREVENTIVE MEDICINE |
| 42 | The first survey of human parasite distribution in Xinjiang | Li Baoshan, Dong Qiang, Tong Suxiang | CHINESE JOURNAL OF PARASITOLOGY AND PARASITIC DISEASES |
| 43 | Epidemiological characteristics of Blastocystis hominis in urban region, southwestern China | Zhang Shunxian, Tian Liguang, Lu Yan, Li Lanhua, Chen Jiaxu, Zhou Xiaonong | Chinese Journal of Zoonoses |
| 44 | Blastocystis hominis: A new understanding of ancient parasite species | Yu Shukan | JIANGXI JOURNAL OF MEDICAL LABORATORY SCIENCES |
| 45 | Investigation of Blastocystis hominis infection in Xi'an city, Shaanxi province | Qiao Jiying, Li Xiaoying, Cai Juan, Zhang Xu, Wei Zhichao, Li Yaqing | [Journal of Tropical Medicine](http://www.wanfangdata.com.cn/perio/detail.do?perio_id=rdyxzz&perio_title=Journal%20of%20Tropical%20Medicine) |
| 46 | Investigation of intestinal parasitic infection in sheep and goats in Anhui Province and adjacent Provinces | Tang Li, Fu Bu Yin, Wang Kai, Chen Miao, Li Hui, Kan Zhenzhen, Li Wenchao, Gu Youfang | Journal of Anhui Agricultural University |
| 47 | The detection and clinical study of children's repeating bellyache and intestinal protozoan infection | Liao Hongqun, Qiu Wei, Wang Ling, Su Shuilian, Wang Huabin, Chen Guifeng, Zhang Ruiqi | JOURNAL OF GANNAN MEDICAL UNIVERSITY |
| 48 | Study on the infection status of Cryptosporidium and Blastocystis hominis in HIV/AIDS patients in Guangxi and the genotyping of Cryptosporidium | Wang Zunfu | Guangxi Medical University |
| 49 | The infection status, genotyping and in vitro drug sensitivity test of Blastocystis hominis in inpatients in two tertiary hospitals in Guangxi | Yan Hui | Guangxi Medical University |
| 50 | Investigation and molecular epidemiological study on the infection of Blastocystis hominis in different populations in my country | Li Lanhua | Chinese Center for Disease Control and Prevention |
| 51 | Molecular epidemiology and population structure of zoonotic intestinal protozoa of black goats in parts of Yunnan | Xie Shichen | Shihezi University |
| 52 | Analysis on the species distribution and population structure of endoamoeba and Blastocystis in Qinghai yak | Ren Mei | Northwest A&F University |
| 53 | Molecular epidemiological investigation and zoonotic risk analysis of four intestinal protozoa on large-scale pig farms in Fujian Province | Zhang Ning | Fujian Agriculture and Forestry University |
| 54 | Study on infection status and subtypes of Blastocystis calves in some areas of Shaanxi | Wang Shasha | Northwest A&F University |
| 55 | Study on infection and genotyping of Blastocystis suis in some areas of Shaanxi | Hu Ruisi | Northwest A&F University |
| 56 | Molecular Epidemiology Investigation and Risk Factor Assessment of Three Types of Intestinal Protozoa of Domestic Black Bears in Heilongjiang Province | Fei Yuchao | Heilongjiang Bayi Land Reclamation University |
| 57 | Study on the infection and population structure of important zoonotic intestinal protozoa in goats in some areas of Shaanxi Province | Song Junke | Northwest A&F University |
| 58 | Molecular Epidemiology of Three Intestinal Protozoa of Some Birds and Mammals in Harbin, Heilongjiang | Li Qiao | Northeast Agricultural University |
| 59 | Study on infection and population structure of blastocystis in some rare wild animals in Qinling Mountains | Hu Xiongfeng | Northwest A&F University |
| 60 | First report of Blastocystis in giant pandas, red pandas, and various bird species in Sichuan province, southwestern China | Deng, L. Yao, J. X. Liu, H. F. Zhou, Z. Y. Chai, Y. J. Wang, W. Y. Zhong, Z. J. Deng, J. L. Ren, Z. H. Fu, H. L. Yan, X. Yue, C. J. Peng, G. N. | Int J Parasitol Parasites Wildl |
| 61 | Prevalence and subtype distribution of Blastocystis in ethnic minority groups on both sides of the China-Myanmar border, and assessment of risk factors | Gong, B. Liu, X. Wu, Y. Xu, N. Xu, M. Yang, F. Tong, L. Zhou, K. Cao, J. Liu, A. Shen, Y. | Parasite |
| 62 | Cross-sectional surveys and subtype classification of human Blastocystis isolates from four epidemiological settings in China | Li, L. H. Zhang, X. P. Lv, S. Zhang, L. Yoshikawa, H. Wu, Z. Steinmann, P. Utzinger, J. Tong, X. M. Chen, S. H. Zhou, X. N. | Parasitol Res |
| 63 | Prevalence of Cryptosporidium, Giardia, Blastocystis, and trichomonads in domestic cats in East China | Li, W. Liu, X. Gu, Y. Liu, J. Luo, J. | J Vet Med Sci |
| 64 | Occurrence of Blastocystis sp. and Pentatrichomonas hominis in sheep and goats in China | Li, W. C. Wang, K. Gu, Y. | Parasit Vectors |
| 65 | Intestinal parasite co-infection among pulmonary tuberculosis cases without human immunodeficiency virus infection in a rural county in China | Li, X. X. Chen, J. X. Wang, L. X. Tian, L. G. Zhang, Y. P. Dong, S. P. Hu, X. G. Liu, J. Wang, F. F. Wang, Y. Yin, X. M. He, L. J. Yan, Q. Y. Zhang, H. W. Xu, B. L. Zhou, X. N. | Am J Trop Med Hyg |
| 66 | First genotyping of Blastocystis in yaks from Qinghai Province, northwestern China | Ren, M. Song, J. K. Yang, F. Zou, M. Wang, P. X. Wang, D. Zhang, H. J. Zhao, G. H. Lin, Q. | Parasit Vectors |
| 67 | Molecular characterization of Blastocystis from pigs in Shaanxi province of China | Song, J. K. Hu, R. S. Fan, X. C. Wang, S. S. Zhang, H. J. Zhao, G. H. | Acta Trop |
| 68 | First genotyping of Blastocystis sp. in dairy, meat, and cashmere goats in northwestern China | Song, J. K. Yin, Y. L. Yuan, Y. J. Tang, H. Ren, G. J. Zhang, H. J. Li, Z. X. Zhang, Y. M. Zhao, G. H. | Acta Trop |
| 69 | Extensive multiparasitism in a village of Yunnan province, People's Republic of China, revealed by a suite of diagnostic methods | Steinmann, P. Du, Z. W. Wang, L. B. Wang, X. Z. Jiang, J. Y. Li, L. H. Marti, H. Zhou, X. N. Utzinger, J. | Am J Trop Med Hyg |
| 70 | A cross-sectional study of intestinal parasitic infections in a rural district of west China | Tang, N. Luo, N. J. | Can J Infect Dis |
| 71 | Co-infection of HIV and intestinal parasites in rural area of China | Tian, L. G. Chen, J. X. Wang, T. P. Cheng, G. J. Steinmann, P. Wang, F. F. Cai, Y. C. Yin, X. M. Guo, J. Zhou, L. Zhou, X. N. | Parasit Vectors |
| 72 | Survey on co-infection with HIV and intestinal parasites in high prevalence areas of HIV/AIDS, China | Tian, L. G. Cheng, G. J. Chen, J. X. Cai, Y. C. Guo, J. Tong, X. M. Liu, Q. Zhou, X. N. | Chinese Journal of Schistosomiasis Control |
| 73 | HIV and intestinal parasite co-infections among a Chinese population: an immunological profile | Tian L.G.Wang，T.P.Lv，S.Wang，F.F.F.Guo，J.Yin，X.M.Cai，Y.C.Dickey，M.K.Steinmann，P.Chen，X. | Infect Dis Poverty |
| 74 | Distribution and genetic diversity of Blastocystis subtypes in various mammal and bird species in northeastern China | Wang, J. Gong, B. Liu, X. Zhao, W. Bu, T. Zhang, W. Liu, A. Yang, F. | Parasit Vectors |
| 75 | Subtype distribution and genetic characterizations of Blastocystis in pigs, cattle, sheep and goats in northeastern China's Heilongjiang Province | Wang, J. Gong, B. Yang, F. Zhang, W. Zheng, Y. Liu, A. | Infect Genet Evol |
| 76 | Epidemiological survey of Blastocystis hominis in Huainan City, Anhui Province, China | Wang, K. X. Li, C. P. Wang, J. Cui, Y. B. | World J Gastroenterol |
| 77 | First record of Leptospira and Blastocystis infections in captive flying squirrels (Trogopterus xanthipes) from Enshi County, China | Xiao, X. Zhou, S. H. Jiang, N. Tian, D. Z. Zhou, Z. M. Zhang, M. Ke, H. Jiang, X. C. Lv, W. L. Gao, Q. H. | Acta Trop |
| 78 | Case-control study of diarrheal disease etiology in individuals over 5 years in southwest China | Zhang, S. X. Yang, C. L. Gu, W. P. Ai, L. Serrano, E. Yang, P. Zhou, X. Li, S. Z. Lv, S. Dang, Z. S. Chen, J. H. Hu, W. Tian, L. G. Chen, J. X. Zhou, X. N. | Gut Pathog |
| 79 | Impact of co-infections with enteric pathogens on children suffering from acute diarrhea in southwest China | Zhang, S. X. Zhou, Y. M. Xu, W. Tian, L. G. Chen, J. X. Chen, S. H. Dang, Z. S. Gu, W. P. Yin, J. W. Serrano, E. Zhou, X. N. | Infect Dis Poverty |
| 80 | Genotyping of Enterocytozoon bieneusi and Subtyping of Blastocystis in Cancer Patients: Relationship to Diarrhea and Assessment of Zoonotic Transmission | Zhang, W. Ren, G. Zhao, W. Yang, Z. Shen, Y. Sun, Y. Liu, A. Cao, J. | Front Microbiol |
| 81 | Ascaris spp. and Capillaria caudinflata infections in captive-bred crested ibis (Nipponia nippon) in China | Zhang, X. Qiao, J. Y. Wu, X. M. Ma, Q. Y. Hu, H. Wang, J. Che, L. F. | Zoo Biol |
| 82 | First report of Blastocystis infections in cattle in China | Zhu, W. Tao, W. Gong, B. Yang, H. Li, Y. Song, M. Lu, Y. Li, W. | Vet Parasitol |
